# Limited Transfer of Working Memory Training to Instrumental Activities of Daily Living in Chronic Stroke Survivors: A Randomized Controlled Trial

**DOI:** 10.3390/pathophysiology32030040

**Published:** 2025-08-22

**Authors:** Daniel Landínez-Martínez, Andres Grisales-Aguirre

**Affiliations:** 1 Faculty of Health Sciences, Universidad de Manizales, Manizales 170001, Colombia; 2 Faculty of Engineering, Universidad de Manizales, Manizales 170001, Colombia; agrisalesa@umanizales.edu.co

**Keywords:** stroke, functional independence, working memory training, randomized controlled trial, neuroplasticity

## Abstract

**Background/Objectives:** Post-stroke cognitive impairment significantly impacts long-term functional outcomes, particularly in instrumental activities of daily living (IADLs). Working memory training (WMT) has emerged as a potential cognitive rehabilitation strategy; however, its transfer to real-world functionality remains unclear. This study evaluated whether adaptive computerized WMT enhances IADLs performance compared to a non-adaptive control condition in chronic stroke survivors. **Methods:** A single-blind, randomized controlled trial was conducted with 50 adults aged 50–79 years, ≥12 months post-ischemic stroke, and diagnosed with a mild neurocognitive disorder. Participants were randomized to adaptive WMT or non-adaptive cognitive training, each completing 25 home-based sessions over 12 weeks via a standardized online platform. Primary outcomes included the Lawton and Brody IADL Scale and the Working Memory Questionnaire (WMQ); secondary outcomes included the Working Memory Index (WMI) from the WAIS-IV. Analyses included frequentist and Bayesian methods. **Results:** Both groups showed significant pre–post improvements in IADL independence and WMI (*p* < 0.05; BF_10_ > 10), with no significant between-group differences on overall IADL outcomes. The adaptive WMT group demonstrated specific gains in WMQ—Storing (*p* = 0.033; BF_10_ = 3.83), while the control group improved in WMQ—Attention and IADL—Assistance Required (*p* = 0.004–0.035; BF_10_ > 6). Bayesian ANOVA indicated that these effects were primarily driven by the interventions, with minimal influence from depressive symptoms or global cognition. **Conclusions:** Adaptive WMT yielded domain-specific cognitive benefits but did not enhance IADL performance beyond non-adaptive training. These findings highlight the limited far transfer of WMT and the importance of designing ecologically valid, multimodal rehabilitation strategies post-stroke.

## 1. Introduction

As of 2021, an estimated 93.8 million individuals worldwide live with the consequences of stroke, corresponding to a crude prevalence rate of 1189 per 100,000 people, with a nearly equal distribution between men and women and 65% of survivors being under the age of 70 [1,2]. Cognitive impairment affects approximately two-thirds to three-quarters of stroke survivors, compromising not only basic activities of daily living (ADLs) but also instrumental activities of daily living (IADLs) such as managing finances, shopping, preparing meals, and handling household tasks. A systematic review and meta-analysis reported a medium association (r = 0.37) between cognitive deficits and limitations in daily functioning, underscoring that impairments in attention, memory, language, and executive functions contribute significantly to long-term disability [3,4].

IADLs serve as critical indicators of functional independence, quality of life, and social participation [5]. Persistent difficulties in IADL performance—documented from three months up to 11 years post-stroke—highlight the need for routine cognitive assessments to enable early detection and personalized rehabilitation strategies [6,7,8,9]. In this context, working memory has emerged as a pivotal cognitive function [10]. A recent study demonstrated that a lower working memory capacity, measured by the Forward Digit Span (FDS) and Backward Digit Span (BDS), is significantly correlated with a greater IADL dependency (*p* = 0.005 for FDS; *p* = 0.010 for BDS). Moreover, survivors without cognitive impairment exhibited markedly lower IADLs scores (0.38 ± 1.071) compared to those with impairment (5.41 ± 5.06, *p* = 0.001) [11]. Complementing these findings, another study underscored that a working memory is essential for maintaining and manipulating information—a process fundamental to functional recovery [12]. Thus, early cognitive screening and targeted interventions aimed at enhancing working memory are imperative for improving IADLs performance, promoting independence, and optimizing long-term rehabilitation outcomes.

Research on working memory training (WMT) in stroke rehabilitation has produced mixed findings, with some studies reporting cognitive improvements but limited evidence supporting gains in IADLs. A systematic review examined cognitive rehabilitation for post-stroke attention deficits and found that, while divided attention showed moderate short-term improvements (SMD = 0.67, *p* < 0.0001), no significant long-term effects were observed on global cognitive function (SMD = 0.16, *p* = 0.41; *n* = 99) or functional independence [13,14]. Similarly, a pilot study demonstrated statistically significant improvements in working memory scores after computerized WMT, but these gains did not translate into broader cognitive or functional benefits [15].

Despite evidence supporting near-transfer effects, the far-transfer of cognitive gains to real-world IADL performance remains unsubstantiated. A systematic review by van de Ven et al. (2016) found that while WMT improved tasks similar to training, there was no evidence of functional gains in everyday activities [16]. Importantly, when active control groups were used, no significant differences emerged in IADLs outcomes. Additionally, a Cochrane review by Das Nair et al. (2016) found small, short-term improvements in self-reported memory function (SMD = 0.36, *p* = 0.01) but no long-term effects on IADLs, mood, or quality of life, suggesting that observed benefits may be temporary or task specific [17].

Several methodological limitations undermine the generalizability of these findings. Many studies had small sample sizes (15; *n* = 20), lacked active control conditions, and failed to conduct long-term follow-ups. Additionally, most studies relied on laboratory-based cognitive assessments rather than ecologically valid IADL measures, limiting their clinical applicability [16]. These limitations highlight the critical need for robust randomized controlled trials (RCTs) with extended follow-ups, larger samples, and validated functional outcome measures to determine whether WMT can contribute meaningfully to post-stroke rehabilitation.

The theoretical foundation for WMT in stroke rehabilitation is anchored in neuroplasticity, cognitive training models, and the challenge of transfer effects. Neuroplasticity, the capacity of the brain to reorganize itself by forming new neural connections, underlies cognitive training interventions [18]. Empirical evidence demonstrates that WMT can induce task-specific changes in functional connectivity, particularly within frontoparietal networks implicated in executive functions [19]. However, while localized neural adaptations occur, the extent to which these modifications translate to functional independence in daily life remains debated [20]. IADLs such as medication management, financial decision-making, and meal preparation, demand the coordinated integration of multiple cognitive and motor domains [21]. While working memory is crucial for maintaining and manipulating information during complex tasks, IADLs performance also requires intact problem solving, motor sequencing, and adaptive decision-making [22]. Evidence suggests that improving isolated cognitive functions does not necessarily lead to gains in real-world functional outcomes, as IADLs depend on domain-general cognitive integration rather than single-domain improvements.

A fundamental limitation in WMT research is the challenge of achieving far-transfer effects, in which cognitive improvements extend beyond the trained task to real-world activities. The ecological validity of traditional WMT paradigms is often limited, as training exercises are decontextualized from everyday challenges [23]. Furthermore, stroke survivors exhibit substantial interindividual variability in baseline cognitive reserve, lesion location, and rehabilitation engagement, affecting training efficacy and transfer potential [24]. Given these constraints, emerging mechanistic models advocate for hybrid interventions combining WMT with real-world functional training to enhance IADLs performance [25]. Studies should focus on designing ecologically valid, multimodal rehabilitation strategies that leverage both cognitive and motor learning principles to optimize post-stroke functional recovery.

The efficacy of WMT in improving functional independence after stroke remains an unresolved question in cognitive rehabilitation research. While prior studies have demonstrated near-transfer effects—where cognitive gains are confined to tasks closely resembling training—the evidence for far-transfer to IADLs is inconsistent and methodologically limited. Given that IADLs require the integration of multiple cognitive and motor domains, the assumption that isolated working memory improvements will generalize to complex real-world tasks remains empirically unverified. Moreover, many existing studies suffer from small sample sizes, inadequate control conditions, and a reliance on laboratory-based cognitive measures with limited ecological validity. Addressing these limitations is imperative to refine rehabilitation strategies and optimize post-stroke functional recovery.

This study employs a rigorously designed randomized controlled trial (RCT) with an active control group, ensuring that observed effects are attributable to WMT rather than nonspecific training factors. Standardized, ecologically valid IADLs assessments will be used to evaluate real-world functional outcomes, with a longitudinal follow-up to assess the persistence of potential benefits.

We hypothesize that, while WMT may enhance task-specific cognitive performance, it will not produce significant improvements in IADL functioning compared to standard rehabilitation. This study will provide critical empirical evidence to determine the translational value of WMT and inform the development of targeted interventions that effectively promote functional independence in stroke survivors.

## 2. Materials and Methods

Study Design

This study employed a single-blind, RCT design comprising two parallel arms: an experimental group receiving working memory training and an active control group. Randomization was performed using a computer-generated sequence by an independent statistician prior to participant enrollment. Allocation was concealed until completion of the baseline assessments to mitigate selection and performance biases. Outcome evaluators were blinded to group assignment and pre-intervention data to ensure objectivity in data collection.

Participants

**Figure 1:**
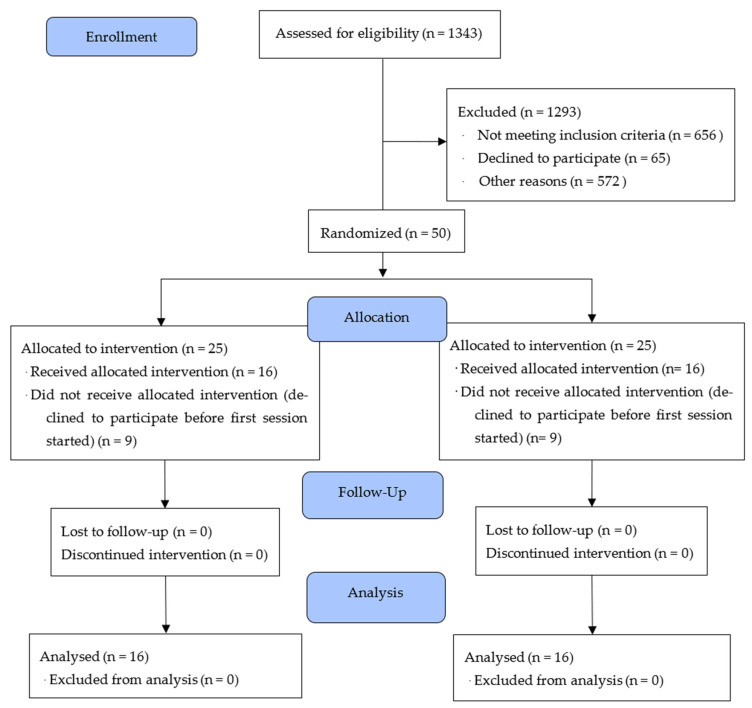
CONSORT-SPI 2018 diagram showing flow of participants through the study, including screening, randomization, and attrition. Of the 50 randomized participants, 18 (36%) withdrew before initiating the intervention (*n* = 9 per group), with no losses to follow-up thereafter. This reduced the final analyzed sample to 32 participants (16 per group) and lowered the achieved statistical power from the planned 0.90 to approximately 0.78 for detecting a medium-sized Group × Time interaction effect (f = 0.25).

Participants were recruited from the Instituto Neurológico de Colombia between October 2021 and November 2023. A total of 1343 stroke survivors were referred by treating clinicians. After applying the inclusion and exclusion criteria, 50 participants were randomized into the trial (see Figure 1 for the CONSORT flow diagram). All participants provided written informed consent, and the study received ethical approval from the institutional review board (Approval Code: 449011-19.02-013).

Inclusion criteria were as follows: (1) age between 50 and 79 years old; (2) history of a first-ever ischemic stroke confirmed via computed tomography (CT) or functional magnetic resonance imaging (fMRI); (3) stroke onset ≥ 12 months prior to enrollment; (4) minimum completion of primary education; and (5) fulfillment of DSM-5 criteria for mild neurocognitive disorder, operationalized using the Montreal Cognitive Assessment (MoCA), with cutoffs adjusted for educational attainment (scores of 21–22 for 5–10 years of education and 23–24 for ≥11 years).

Additional eligibility requirements included access to a computer with a stable internet connection, availability of a primary caregiver during online sessions, and no concurrent participation in formal cognitive or physical rehabilitation programs. Exclusion criteria included the following: (1) significant motor impairment of the dominant upper limb preventing use of digital interfaces; (2) clinically significant depressive symptoms (Yesavage Geriatric Depression Scale score ≥ 10); (3) history of major psychiatric disorders; (4) aphasia or communication disorders interfering with assessment; (5) diagnosis of major neurocognitive disorder; and (6) inability to provide informed consent. Due to substantial heterogeneity and incomplete neuroimaging reports, data on lesion location and volume were not included in this analysis.

Assessment Procedures

All assessments were conducted via secure video conferencing using the Google Meet platform. Evaluations were supervised in real time by a licensed neuropsychologist. The assessment protocol was divided into two sessions to minimize fatigue and maximize adherence. In the first session, the following instruments were administered: a standardized sociodemographic interview, the Instrumental Activities of Daily Living scale (Neuronorma adaptation), and the Working Memory Questionnaire. The participant, caregiver, and neuropsychologist joined a secure online session, during which the caregiver was instructed to support only technical logistics (e.g., screen sharing) and to refrain from providing cognitive assistance unless explicitly instructed. The second session focused on the neuropsychological assessment and included the administration of the Working Memory Index. Standardized instructions and demonstration trials were provided before task initiation. The neuropsychologist observed the participant’s screen during task completion to ensure protocol compliance and recorded their performance in real time. The caregiver was again present to assist with screen sharing but was asked to remain passive during task execution. All assessments were conducted in a controlled digital environment, with consistent protocols across participants to ensure standardization and reduce measurement variability.

Intervention

Participants assigned to the intervention group received a fully automated, adaptive computerized working memory training program, while those in the control group completed a non-adaptive version of the same program. Both versions were accessed through a secure online platform https://braining.me (accessed on 4 March 2024) and developed by the research team using identical user interfaces, visual design, and task structures to control for engagement, expectancy, and usability.

Each training protocol comprised six computerized tasks specifically targeting working memory processes, including updating, maintenance, and manipulation. Tasks provided both trial-by-trial feedback and cumulative performance summaries. The key distinction between conditions was the adaptivity of the training protocol: the adaptive version employed a real-time difficulty adjustment algorithm that dynamically increased task complexity based on individual performance (ranging from 1 to 15 stimuli), thereby maintaining an optimal challenge level. In contrast, the non-adaptive control condition used a fixed, low-difficulty threshold (1 to 5 stimuli), which did not change throughout the training period, thus minimizing cognitive load and limiting the potential for skill acquisition.

Participants were instructed to complete daily training sessions of approximately 50 min each, targeting a total of 25 sessions over a 12-week period. Each session resumed from the final difficulty level attained on the previous day to ensure continuity in training progression. Training was conducted remotely, with participants using personal computers in their home environment.

To monitor adherence and identify potential barriers, participants received weekly follow-up calls from a trained research assistant. During these structured interviews, participants reported perceived cognitive, emotional, and social changes, as well as technical challenges encountered. Technical support was provided as needed to ensure consistent access and minimize dropout.

Outcome Measures

All outcome measures were collected at two time points: baseline (pre-intervention) and post-intervention (within 12 weeks of completing the training program). Assessments were administered in one or two sessions, depending on participant availability and fatigue levels, and were supervised remotely by a licensed neuropsychologist.

Primary Outcome Measures

The primary outcome was instrumental activities of daily living (IADLs), assessed using two validated instruments.

Lawton and Brody Instrumental Activities of Daily Living Scale: This scale evaluates functional competence across domains such as medication management, financial handling, transportation, and housekeeping. Both total scores and item-level responses were analyzed to capture granular changes in specific IADL components [26].

Working Memory Questionnaire: A self-report measure designed to capture everyday difficulties related to working memory in daily contexts [27].

All raw scores were adjusted for age and education based on available normative data. Importantly, neither the Lawton IADL scale nor the Working Memory Questionnaire was included in the training protocol, ensuring that outcome data reflected transfer effects rather than task-specific improvements.

Secondary Outcome Measures

Secondary outcomes focused on working memory capacity, assessed using the following performance-based neuropsychological tasks.

Working Memory Index: Derived from standardized subtests of the Wechsler Adult Intelligence Scale-IV (WAIS-IV), assessing verbal working memory span and manipulation [28].

See Table 1 for an overview of all cognitive tasks, scoring procedures, and associated outcome variables.

Background Measures

To ensure that all participants met the inclusion criteria regarding cognitive and affective status, two standardized screening instruments were administered prior to randomization.

Montreal Cognitive Assessment (MoCA): This brief cognitive screening tool was used to exclude participants with a probable major neurocognitive disorder or dementia [29]. The MoCA has demonstrated adequate internal consistency in older adult populations (Cronbach’s α = 0.75) and provides a global index of cognitive functioning, including attention, memory, executive function, and visuospatial abilities.

Geriatric Depression Scale—Short Form (GDS-15): To exclude clinically significant depressive symptoms, the GDS-15 was administered [30]. This self-report measure is widely used in geriatric populations and has demonstrated an acceptable internal reliability (Cronbach’s α = 0.72). A cutoff score of ≥10 was used as the exclusion criterion in accordance with validated thresholds for moderate to severe depression.

These background measures ensured that cognitive impairment was consistent with a diagnosis of a mild neurocognitive disorder and not confounded by unrecognized dementia or depression.

Sample Size Determination

A priori sample size estimation was conducted using GPower 3.1 [31] for a repeated-measures ANOVA with two groups (adaptive vs. non-adaptive training) and two time points (pre- and post-intervention). Parameters were set to α = 0.05, power (1 − β) = 0.90, and an effect size of f = 0.25 (partial η^2^ ≈ 0.058; equivalent to d ≈ 0.50), reflecting medium effects reported in prior post-stroke cognitive training studies [13,17]. The analysis indicated a minimum total sample size of 30 participants (15 per group) to detect a statistically significant Group × Time interaction. The GPower for this design specifies total, not per-group, sample size requirements. To allow for anticipated attrition, we randomized 50 participants (25 per group). Following exclusions and dropouts, 32 participants (16 per group) completed the intervention and were included in the final analysis, yielding an achieved power of approximately 0.78 for f = 0.25.

Randomization and Blinding

Participants were randomized to either the intervention group (adaptive working memory training) or the active control group (non-adaptive training) using a computer-generated sequence produced by Research Randomizer software. Randomization occurred immediately following telephone screening and prior to accessing participants’ medical records to minimize allocation bias. In cases where a subsequent medical review revealed non-eligibility based on inclusion/exclusion criteria, the participant was informed of their exclusion and replaced with a new recruit maintaining the integrity of the randomization process.

Group assignment was stratified to minimize imbalances across critical baseline characteristics, including age, sex, years of education, level of computer experience, and MoCA scores. Importantly, participants were not informed of the adaptive nature of the intervention group; instead, they were told that the study was comparing two types of computerized cognitive training programs.

To reduce bias, outcome assessors were blinded to group assignment. Allocation was concealed using participant ID codes managed by the research coordinator, and assessors were not involved in the training sessions. At the conclusion of randomization, 25 participants had been assigned to each group.

Statistical Analysis

Before and after treatment, the patients’ performance was evaluated by comparing the mean scores obtained in the Lawton Instrumental Activities of Daily Living, Working Memory Questionnaire, and the Working Memory Index. Data were analyzed using RStudio v. 1.4.1106, and the level of significance was set at *p* < 0.05 for all tests. The normality of the data was checked using the Shapiro–Wilks test. Descriptive statistics were calculated as a mean or median (with standard deviation, interquartile range), while categorical variables were presented as percentage frequencies. A Student’s *t*-test and Mann–Whitney U test were used to test the significant differences in gains between the two groups. The effect size (Cohen’s d) was calculated to determine the group difference for each outcome measure. Cohen suggested that *d* = 0.2 be considered a ‘small’ effect size, 0.5 represents a ‘medium’ effect size, and 0.8 a ‘large’ effect size.

Mixed-Design ANOVA

In addition to the planned between- and within-group comparisons, a two-by-two mixed-design ANOVA was conducted for all primary outcome measures to directly test the group-by-time interaction. The groups were the adaptive and the control, and the times were pre- and post. Both frequentist and Bayesian frameworks were applied. The assumptions of normality (Q–Q plots) and homogeneity of variances (Levene’s tests) were examined, and where appropriate, effect sizes (partial ω^2^) were reported. Although this analysis was performed for all primary outcomes, a statistically significant interaction emerged only for the WMI variable. To examine the potential influence of clinical characteristics within the same framework, we repeated the mixed ANOVA, including age as a between-subjects covariate and adding single comorbidity factors (hypertension 0/1 and diabetes 0/1) one at a time to the between-subjects panel. In each case, we evaluated the Time × Factor and Group × Time × Factor terms while retaining the Group × Time contrast. Stroke etiology was ischemic in all participants and could not therefore be modeled. The above assumptions were checked, and estimated marginal means with Holm-adjusted comparisons are reported where relevant.

Bayesian Analysis

In addition to traditional null hypothesis significance testing (NHST), Bayesian analyses were conducted to quantify the strength of evidence for group differences and the probability of the null hypothesis given the data. Bayesian independent samples t-tests were computed using the Bayes Factor package, and R. Bayes Factors (BF_10_) were interpreted using the guidelines where values between 1 and 3 indicate anecdotal evidence, 3–10 moderate evidence, and values >10 strong evidence for the alternative hypothesis [32].

## 3. Results

The flow of participants through the study can be seen in the CONSORT-SPI 2018 diagram (Figure 1).

Baseline Characteristics

Out of 1343 potential participants who were screened, 50 met all inclusion and exclusion criteria and were randomized (25 per group). Eighteen participants (36%) withdrew before starting the intervention (*n* = 9 per group), most commonly due to loss of interest or scheduling conflicts. No participants were lost to follow-ups after initiating the training. This attrition reduced the sample from the planned 50 to 32 completers (16 per group) and lowered the achieved statistical power from the target 0.90 to approximately 0.78 for detecting a medium-sized Group × Time interaction effect (f = 0.25). Figure 1 presents the CONSORT flow diagram, including detailed reasons for dropout.

Prior to training, the two groups did not differ in age, educational level, sex, or baseline cognitive functioning (see Table 2 for scores and statistics). The active control group included 16 participants, consisting of 10 men (62.5%) and 6 women (37.5%), ranging in age from 52 to 67 years (M = 59.4, SD = 4.9). Years of formal education varied between 5 and 17 years (M = 11.4, SD = 3.2). Time since ischemic stroke ranged from 11 to 112 months (M = 41.8, SD = 25.7). Vascular risk factors included hypertension in seven participants (43.7%) and diabetes in one participant (6.25%).

The experimental group consisted of 16 participants as well, comprising seven men (43.7%) and nine women (56.2%) aged between 50 and 70 years (M = 62.1, SD = 6.2). Years of education ranged from 5 to 17 (M = 11.3, SD = 4.6). Time since the cerebrovascular event ranged from 24 to 127 months (M = 70.2, SD = 36.1). Hypertension was reported in seven participants (43.7%) and diabetes in four (25%).

Mood and Cognitive Screening

Mood status was assessed using the Geriatric Depression Scale. In both groups, at least 75% of participants scored within the non-depressed range, while the remaining 25% showed scores consistent with mild depressive symptoms. In the active control group, scores ranged from 0 to 8 (M = 3.1, SD = 3.0). In the experimental group, scores ranged from 0 to 5 (M = 1.8, SD = 1.4), suggesting an overall lower depressive symptomatology. Global cognitive functioning was evaluated using the Montreal Cognitive Assessment (MoCA), adjusted for years of education. Both groups met the criteria for mild neurocognitive disorders, with comparable performance: the control group scored M = 24.1 (SD = 1.6) and the experimental group M = 23.6 (SD = 0.8). These results indicate a relatively preserved cognitive profile consistent with the inclusion criteria.

The following section presents comparisons of the study variables using non-parametric tests, supported by a Bayes Factor analysis. The first set of comparisons pertains to baseline measurements, with descriptive and inferential statistics summarized in Table 2. The table reports descriptive values (mean, standard deviation, standard error, and coefficient of variation) for each variable at the initial assessment, along with the results of the Mann–Whitney U test, statistical significance (S.S), and the Bayes Factor (BF_10_).

Baseline comparisons revealed no statistically significant differences between the experimental and control groups across any of the measured variables (*p* > 0.05; see Table 2). All Bayes Factor values (BF_10_ < 1) consistently favored the null hypothesis, indicating stronger evidence for the absence of group differences prior to the intervention. Descriptive statistics further demonstrated comparable means and coefficients of variation between groups, suggesting a homogenous distribution of scores across cohorts. These findings support the baseline equivalence and provide a robust foundation for evaluating post-intervention outcomes.

Similarly, a comparison of the variables between the control group and the experimental group was conducted for the second administration of the scales. The results are presented in Table 3.

As shown in Table 3, no statistically significant differences were observed between the control and experimental groups in a majority of the variables following the intervention (*p* > 0.05). The corresponding Bayes Factors (BF_10_), which were either close to or below one, further support the null hypothesis, indicating an absence of group-level differences. An exception was observed for the WMI variable, which yielded a statistically significant effect (*p* = 0.015) and a BF_10_ of 2.412, suggesting moderate evidence in favor of the alternative hypothesis. The mean score in the experimental group (103.94) exceeded that of the control group (93.69), reflecting a marked improvement in this domain attributable to the intervention.

To formally test whether the magnitude of change from pre- to post-intervention differed between groups, a two-by-two mixed ANOVA (group: adaptive versus control; time: pre- versus post) was conducted on the WMI scores. The analysis revealed a significant main effect of time (F(1, 30) = 46.60, *p* < 0.001) and a significant Group × Time interaction (F(1, 30) = 11.75, *p* = 0.002). These results indicate that the experimental group exhibited greater pre–post improvement than the control group. The between-subjects group effect was also significant (F(1, 30) = 4.95, *p* = 0.034, and ω^2^ = 0.060). Descriptive statistics showed that the experimental group increased from 92.63 ± 8.54 to 103.94 ± 11.51, while the control group increased from 89.94 ± 7.38 to 93.69 ± 7.04. A Bayesian repeated-measures ANOVA corroborated these results. The model that included the Group × Time interaction was the most probable (P(M|data) = 0.905; FB_M = 38.14). This provides strong evidence that the pre–post change was greater in the experimental group. Assumption checks indicated acceptable normality and homogeneity (Levene’s test: pre-, *p* = 0.526; post, *p* = 0.050; and Q–Q plot showed residuals to be approximately normal).

Similarly, to consider how age and clinical factors might influence training-related change, we examined potential moderators using a repeated-measures ANOVA. When age was entered as a between-subjects covariate, the Group × Time effect on WMI remained significant (F = 15.85, *p* < 0.001, and partial η^2^ = 0.353). However, the Time × Age term was not significant (F = 4.10, *p* = 0.052), suggesting that age was not responsible for the observed effect. Hypertension (0/1) significantly moderated the pre–post difference (Group × Time × hypertension: F(1, 28) = 5.57, *p* = 0.026, and partial η^2^ = 0.166). Gains were larger in the adaptive group among hypertensive participants (adaptive = +15.57 vs. control = +2.57) and smaller, yet still present, in non-hypertensive participants (adaptive = +8.00 vs. control = +4.67). By contrast, diabetes did not moderate change (Group × Time × Diabetes: F(1, 28) = 0.06, *p* = 0.801; Time × Diabetes: F = 0.65, *p* = 0.427). All participants had an ischemic stroke, so the etiology could not be modeled. Assumption checks were acceptable (Levene’s and Q–Q).

Following between-group comparisons, within-group analyses were conducted to evaluate pre- to post-intervention changes for each group independently. Table 4 presents the results of these paired comparisons, including the Wilcoxon signed-rank test statistic (W), z-values, corresponding *p*-values, and Bayes Factors (BF_10_) for each outcome measure.

As shown in Table 4, the variable IADL-I increased from 9.688 to 12.563 (W = 9.500, z = −2.699, and *p* = 0.007), with a Bayes Factor (BF_10_) of 34.076, indicating strong evidence in favor of the alternative hypothesis. In contrast, the variable WMQ (Storing) showed a decrease from 15.375 to 12.438 (W = 98.000, z = 2.158, and *p* = 0.033), with a BF_10_ of 3.827, which reflects moderate evidence supporting a significant change. Additionally, the variable WMI exhibited a notable increase from 92.625 to 103.938 (W = 3.000, z = −3.361, and *p* < 0.05), accompanied by a markedly high BF_10_, indicating very strong evidence for a substantial effect.

Conversely, the remaining variables IADL-AR, IADL-D, WMQ (Attention), WMQ (Executive), WMQ (Full Scale), did not reach statistical significance (*p* > 0.05), and their BF_10_ values remained below the conventional threshold of three, suggesting insufficient evidence to reject the null hypothesis in these cases.

When conducting the same comparison for the control group, the results are presented in Table 5.

The results presented in Table 5 reveal statistically significant changes in several of the analyzed variables. Specifically, the IADL-I subscale showed an increase in the mean score from 10.188 (SD = 3.619) to 12.563 (SD = 2.476). The associated test statistics (W = 0.000, z = −3.059, *p* = 0.002, and BF_10_ = 134.676) provide very strong evidence in favor of a significant time-related effect on this variable. Similarly, the IADL-AR subscale showed a reduction from 2.688 (SD = 2.938) to 0.900 (SD = 1.197) (W = 21.000, z = 2.201, *p* = 0.035, and BF_10_ = 6.687), indicating a statistically meaningful decrease. The WMQ (Attention) score also decreased from 13.063 (SD = 6.005) to 9.250 (SD = 6.191) (W = 111.500, z = 2.925, *p* = 0.004, and BF_10_ = 77.418), suggesting a notable post-intervention effect. In the case of WMQ (Full Scale), the score dropped from 36.313 (SD = 14.988) to 29.813 (SD = 15.510) (W = 102.000, z = 2.385, *p* = 0.018, and BF_10_ = 3.357). Additionally, the WMI dimension showed an increase from 89.938 (SD = 7.380) to 93.688 (SD = 7.040) (W = 6.500, z = −2.550, *p* = 0.012, and BF_10_ = 21.669), indicating a statistically significant improvement.

In contrast, no significant pre- to post-intervention changes were observed in the IADL-D, WMQ (Storing), and WMQ (Executive) subscales, as their *p*-values exceeded the conventional alpha threshold (*p* > 0.05), and their BF_10_ values remained below the cutoff typically used to indicate substantial evidence for an effect (i.e., BF_10_ < 3).

A comparative analysis between the experimental and active control groups revealed that both showed statistically significant improvements in the IADL-I and WMI subscales, suggesting that both cognitive training modalities may positively influence these domains. However, distinctive patterns emerged across groups: the experimental group exhibited a significant reduction in WMQ (Storing) (*p* = 0.033, BF_10_ = 3.827), whereas the active control group demonstrated significant improvements in IADL-AR (*p* = 0.035, BF_10_ = 6.687) and WMQ (Attention) (*p* = 0.004, BF_10_ = 77.418)—effects that were not significant in the experimental condition. Additionally, the WMQ (Full Scale) score decreased significantly in the control group (*p* = 0.018, BF_10_ = 3.357), whereas the same outcome showed only a trend toward significance in the experimental group (*p* = 0.062, BF_10_ = 2.279).

These findings suggest a differential pattern of change across groups: although both interventions were associated with significant improvements in specific cognitive domains, each appears to exert its effect on distinct functional dimensions. This highlights the importance of considering the nature and specificity of cognitive training strategies when evaluating their impact on post-stroke cognitive recovery.

To determine whether the differences observed between pre-test and post-test scores in the assessed variables can be attributed solely to the intervention or whether they may also be explained by the influence of covariates such as depressive symptoms (Depression Yesavage Test) or global cognitive performance (MOCA Test), a Bayesian repeated-measures analysis of variance (ANOVA) was conducted. This methodological approach allowed for the quantitative evaluation of multiple explanatory models, enabling direct comparisons between models that include or exclude the aforementioned covariates.

As an initial step, we examined whether the pre-test–post-test difference in the IADL-I variable, for both the experimental and control groups, was influenced by depressive symptoms as a covariate or whether the observed change could be attributed exclusively to the intervention. The results of this analysis are presented in Table 6.

Bayesian analysis results indicate that, within the experimental group, the model including only the effect of time was the most probable (P(M|data) = 0.506), with very strong evidence (BF = 17.237) and minimal contributions from depressive symptoms (Depression Yesavage Test, BF = 0.860). In contrast, in the control group, although a strong effect of time was also observed (BF = 14.531), the most probable model included depression as a covariate (P(M|data) = 0.656; BF = 2.314), suggesting that other factors may partially account for the observed differences.

Additionally, the Bayesian analyses conducted to evaluate the effect of cognitive performance (MOCA Test) on the dependent variable WMI showed that, in both the experimental and control groups, the model including both time and the MOCA Test was the most probable. However, the specific contribution of the MOCA Test was substantially greater in the control group (BF = 5.559, moderate to strong evidence) compared to the experimental group (BF = 1.149, weak or anecdotal evidence). These findings suggest that, although the intervention was a key determinant in the improvement of WMI, cognitive differences measured by the MOCA Test played a more relevant role in explaining the observed changes within the active control group. These results are summarized in Table 7.

In the control group, the Bayesian repeated-measures analysis conducted to evaluate the effect of time and the depression covariate (Depression Yesavage Test) on the attention variable WMQ (Attention) indicated that the most probable model included both factors (P(M|data) = 0.503). The effect of time was extremely robust (BF = 21.567), reflecting a significant difference between the pre-test (M = 13.06, SD = 6.01) and post-test (M = 9.25, SD = 6.19) scores, which was primarily attributable to the effect of time or the intervention itself, although a specific contribution of depression was also identified (BF = 1.113). The model exhibited a high explanatory capacity, with a mean R^2^ of 72.7% (95% CI: 54.2–84.3%).

Regarding the IADL-AR variable in the control group, the Bayesian analysis revealed that the most probable model was the one including only the depression covariate, with a posterior probability of 0.399. The contribution of this covariate yielded anecdotal evidence (BF = 1.100), while the effect of time was negligible (BF = 0.368), consistent with the stability observed in the pre-test and post-test means (both M = 1.75). This model also showed a high explanatory capacity, with a mean R^2^ of 76.1% (95% CI: 47.9–90.8%), suggesting that depressive symptoms may be weakly related to individual variation in IADL-AR scores, although not to changes over time or in response to the intervention.

Moreover, the Bayesian analysis for the same variable IADL-AR indicated that the most probable model was the null model, with a posterior probability of 0.391. Neither the cognitive covariate (MOCA TOT) (BF = 0.786) nor the effect of time (BF = 0.427) provided sufficient evidence to explain the significant variance in this variable. This finding aligns with the observed identical means in the pre-test and post-test (both M = 1.75). The overall explanatory capacity of the model remained high (mean R^2^ = 78.9%, 95% CI: 56.1–91.7%), which may indicate that unmeasured individual factors contributed substantially to the observed variance.

For the remaining variables included in the study, neither the depression covariate nor the cognitive measure (MOCA) demonstrated a significant contribution to explaining the changes observed between the pre-test and post-test assessments, either within the control or experimental groups. In all these cases, the most probable models were those including only the effect of time, supported by strong Bayes Factors (BF), whereas the effects of the covariates showed weak or anecdotal evidence. These findings suggest that the observed differences in the assessed variables are primarily attributable to the intervention itself or the passage of time, rather than to individual factors such as depression or cognitive performance.

## 4. Discussion

The present study revealed that both adaptive working memory training (WMT) and active control (non-adaptive) training produced significant gains in objective working memory capacity (WMI) and instrumental activities of daily living (IADL) independence. Specifically, participants in both groups showed robust pre–post improvements in WMI and the IADL—Independent (IADL-I) subscale (WMI increased from ~90 to ~94; IADL-I from 10.2 to 12.6) with very strong statistical (*p* < 0.05) and Bayesian evidence (BF_10_ ≫ 10) (Table 5). Both groups improved on WMI, but the mixed-design ANOVA confirmed that the adaptive training group experienced a significantly greater gain. Converging frequentist and Bayesian evidence supported this pattern, reinforcing the idea that the adaptive condition produced a larger improvement in the objective working memory capacity. However, this interaction effect was not observed for the other outcome measures. This suggests that the advantage of adaptivity is domain-specific rather than general across all cognitive or functional domains. Sensitivity checks within the same mixed-ANOVA framework revealed that age was not responsible for the WMI effect, and diabetes did not moderate the effect. However, hypertension was associated with a larger adaptive group gain. Given the sample size, this moderation is considered exploratory. These parallel improvements suggest that any structured cognitive training can enhance test-based working memory performance and basic IADL independence in post-stroke patients, consistent with prior findings that engaging cognitive exercises can bolster one’s capacity under practice conditions [33].

However, the pattern of change diverged between groups in ways that hint at differential mechanisms. The adaptive WMT group (experimental condition) showed a significant reduction in the self-reported Working Memory Questionnaire (WMQ) Storing subscale (*p* = 0.033, BF_10_ ≈ 3.8), whereas the control group improved on the IADL—Assistance Required (IADL-AR) subscale and the WMQ Attention subscale (both *p* ≈ 0.035–0.004, BF_10_ > 6). In other words, only adaptive training was associated with gains in the “Storing” aspect of working memory (perhaps reflecting the enhanced updating of information), while the non-adaptive training primarily improved participants’ attention-related memory complaints and reduced their need for assistance in daily tasks. Notably, the WMQ Full Scale score (overall subjective WM complaints) declined significantly in the control group (*p* = 0.018, BF_10_ ≈ 3.36) but only showed a trend in the experimental group. Thus, although both groups objectively improved on WMI, their subjective reports differed: the control group reported broad improvements in WM problems, whereas the adaptive group reported improvements specifically in storage/updating.

This dissociation between objective and subjective memory measures is striking. It suggests that objective test gains did not translate uniformly into perceived real-world memory function. The adaptive group’s enhanced WMI (↑93.7 vs. 89.9) occurred alongside relatively modest changes in the WMQ total and subscales. Conversely, the control group’s subjective WM complaints improved more than the adaptive group’s despite similar WMI gains. Such a mismatch echoes the well-known finding that cognitive training often yields a limited far transfer to everyday cognition: meta-analytic reviews conclude that working memory training produces a reliable near-transfer (on similar tasks) but no convincing broad “real-world” cognitive benefits [23,34]. In our sample, patients’ insights may also play a role—some stroke patients (especially with right-hemisphere lesions) exhibit anosognosia or reduced awareness of deficits [35]. This could partly explain why subjective WMQ scores did not parallel objective gains or why the control group (perhaps due to expectancy or task differences) felt greater improvement.

Importantly, pre–post gains in IADLs and WMI were largely driven by the interventions themselves, not the baseline mood or cognition. Bayesian repeated-measures ANOVAs (with covariates) indicated that in the adaptive group, improvements in IADL–I were best explained by time (training) alone (BF_10_ ≈ 17.2 for time; BF_10_ ≈ 0.86 for depression). In the control group, time also had a strong effect (BF_10_ ≈ 14.5), but the most probable model included depressive symptoms (Yesavage Test) as a covariate (BF_10_ ≈ 2.31). Similarly, while WMI gains were driven by training in both groups, baseline global cognition (MoCA) played a much larger role in the control group (BF_10_ ≈ 5.56, moderate evidence) than in the adaptive group (BF_10_ ≈ 1.15, anecdotal). In practical terms, this implies that the adaptive training’s effects on memory were relatively independent of patients’ moods or cognitive status, whereas in the non-adaptive group, participants who were already higher functioning (or less depressed) benefited more. Thus, covariates were generally weak contributors, except that baseline depression and cognition modestly modulated outcomes in the control group.

These findings fit within broader theories of neuroplasticity and cognitive training. According to a recent study, effective rehabilitation depends on experience-dependent plasticity: improvements arise from specific, intense, salient, and repetitive practice [18]. In our study, both training regimens involved repetition and some level of difficulty adjustment, but only the adaptive WMT continuously scaled the task challenge to participant performance. This adaptive “optimal challenge” may have preferentially engaged the neural circuits underpinning working memory updating. In this sense, the adaptive group’s specific gain in WMQ (Storing) may reflect a strengthening of dynamic memory updating mechanisms, consistent with the specificity principle of training-induced plasticity [33]. By contrast, the control regimen (with fixed or less challenging tasks) perhaps served more as a general mental exercise, yielding broader subjective improvements (e.g., in attention complaints) but fewer gains in complex updating. Notably, the authors found that adaptive versus non-adaptive training made little difference: exposure to variable task difficulty alone was sufficient to boost working memory performance, with no additional far transfer [20]. Our results partially concur, with both groups improving the objective WMI equally, suggesting that adaptivity, per se, was not required for *baseline* gains. However, the pattern of transfer differed, implying that adaptivity may shape which cognitive processes benefit.

The transfer of cognitive training to everyday function remains controversial. Some recent trials in stroke report that computerized cognitive training improves neuropsychological scores but yields minimal functional gains [36]. For example, this study found significant improvements in processing speed and language tasks after adaptive training, yet no change in basic or instrumental ADLs [36]. In contrast, our results showed a sizeable IADL–I improvement in both groups (mean ∆ ≈ +2.4 points) and a large reduction in the need for assistance (IADL–AR ↓1.8). This discrepancy could arise from differences in patient chronicity, intensity of spontaneous recovery, or the specific IADL measure used. It may also reflect our finding that even non-adaptive cognitive exercises can bolster everyday independence in the short term. Nevertheless, our improvements in IADL coexist with the broader literature cautioning that gains on ‘brain training’ tasks often do not translate into far transfer [23,34]. In particular, recent findings emphasize that most training benefits remain task-specific, with little evidence for enhancements in general cognition or daily life performance [23]. Our data mirror this: aside from IADL–I, other daily life measures (e.g., IADL—Dependent) did not change, and only certain IADL subscales improved (differently by group). Thus, even where we see promising IADL results, they should be interpreted cautiously in light of transfer limitations.

From a clinical standpoint, these findings highlight both the potential and challenges for cognitive rehabilitation after stroke. On one hand, the adaptive WMT appeared to tap into updating-related plasticity, consistent with the idea that training difficulty and task relevance can guide the recovery of specific cognitive mechanisms [18]. On the other hand, improvements did not generalize uniformly, underscoring that cognitive gains do not automatically resolve functional deficits. Stroke recovery is complex and highly individual: lesion location/extent predicts which cognitive and ADL abilities are affected [36], and personal factors (motivation, insight, and mood) also modulate outcomes. A “one-size-fits-all” program is unlikely to fully address this heterogeneity [36]. In line with this, recent reviews advocate for multimodal, ecologically valid interventions: for example, combining cognitive exercises with goal-oriented ADL tasks or aerobic exercise to maximize plasticity (e.g., enriching the training environment) [18,20,36]. Our data suggest that future programs should incorporate such principles—for instance, adapting tasks based on patient progress, varying stimuli to promote generalization, and explicitly linking training to daily activities. Notably, despite some gains here, key WMQ subscales (e.g., Executive) and the IADL—Dependent score showed no change, hinting that either the training dose was insufficient, or the measures lacked sensitivity.

Another methodological consideration is that our inclusion criteria required a diagnosis of a mild neurocognitive disorder post-stroke, confirmed via the MoCA, but we did not stratify participants according to baseline working memory impairment. This decision was intentional to preserve ecological validity, reflecting the heterogeneity of patients referred to outpatient cognitive rehabilitation services, where working memory deficits often co-occur with other cognitive sequelae. Nevertheless, such heterogeneity may have attenuated the potential magnitude of working memory training effects, as previous studies have reported larger gains in individuals with clearly documented deficits in the targeted domain [15]. Future randomized controlled trials could address this by adopting enrichment strategies or pre-stratification based on baseline working memory performance to determine whether treatment effects are moderated by initial impairment severity.

Finally, a key limitation was the attrition rate (36%), which reduced our achieved statistical power from the planned 0.90 to approximately 0.78 for the target medium effect size (f = 0.25). While this power remains within the range considered acceptable for detecting medium effects, it increases the risk of a Type II error, particularly for outcomes where effect sizes were smaller than anticipated. Future studies should incorporate strategies to minimize early withdrawal and consider oversampling accordingly.

## 5. Conclusions

Several caveats temper our conclusions. Sample size was modest and attrition nontrivial, which reduces the statistical power and raises the risk of Type I/II errors. The training duration and intensity (number of sessions) were limited; a longer or more intensive regimen might produce larger or more durable effects. Our IADL instrument may have ceiling or floor effects (e.g., low baseline IADL-AR scores in many patients), possibly obscuring subtler changes. Likewise, the WMQ is a subjective questionnaire; responses can be biased by patient insight or demand characteristics. Finally, although we included covariates (depression, MoCA), other factors (e.g., lesion site, fatigue) were not controlled and could influence outcomes. Future work should employ larger, more diverse samples, include active control tasks closely matched in engagement, and assess long-term follow-ups to confirm the persistence of gains.

In sum, both adaptive WMT and non-adaptive cognitive practice yielded improvements in working memory performance and IADL independence post-stroke, but adaptive training led to changes suggestive of enhanced memory updating processes. This finding was further supported by the mixed-design ANOVA, which confirmed that the adaptive training group showed a significantly greater improvement in WMI from pre- to post-testing than the other outcome measures. This advantage was not accounted for by age or by diabetes, and stroke etiology was constant (ischemic), whereas an exploratory analysis suggested a larger benefit among participants with hypertension. The dissociation between objective and subjective outcomes underscores the need to bridge lab-based gains with real-world function. Designing more ecologically valid training programs—for example, by integrating cognitive tasks with daily living activities, providing strategy instruction, or combining mental with physical exercises—may promote transfer and engage neuroplasticity more fully. Such hybrid interventions, guided by principles of intensity, variety, and relevance, hold promise for improving functional recovery. Ultimately, our findings highlight that while cognitive training can harness brain plasticity after stroke, its effectiveness depends critically on training specificity and the match between tasks and patients’ everyday needs.

## Figures and Tables

**Table 1:** Tasks used for every domain.

Domain	Task	Outcome Measure
Primary outcome measure		
IADL	Lawton Instrumental Activities of Daily Living: (Cronbach’s α = 0.94).	Number of activities performed: - Independent (1–14) - Assistance Required (1–14) - Dependent (1–14)
	e.g., using the telephone	- Independent (3) - Assistance Required (2) - Dependent (1)
	Working Memory Questionnaire: (Cronbach’s α = 0.89). e.g., when you shop, do you often spend more than the budget you set for yourself?	30 questions Each question was rated on a five-point Likert-type scale, ranging from 0 (“no problem at all”) to 4 (“very severe problem in everyday life”). Three sub- scores were computed for each of the three domains (maximal score 40 for each), as well as a total score (out of 120). Higher scores corresponded to more difficulties/complaints.
Secondary outcome measure		
Working memory	Working Memory Index: Arithmetic task, forward, backward and sequencing digit span. (Cronbach’s α = 0.94). e.g., there are 25 gum tablets in each package. How many tablets are there in 8 packages?	Age-corrected z-scores of total number of correct items

**Table 2:** Descriptive and comparative statistics of baseline variables.

Variables	Group	Mean	SD	SE	C.V	*U*	S.S	BF_10_
IADL-I	Control	10.188	3.619	0.905	0.355	144.500	0.543	0.370
	Experimental	9.688	3.516	0.879	0.363			
IADL-AR	Control	2.688	2.938	0.734	1.093	123.500	0.878	0.358
	Experimental	3.000	3.651	0.913	1.217			
IADL-D	Control	1.125	1.893	0.473	1.683	111.000	0.504	0.408
	Experimental	1.313	1.537	0.384	1.171			
WMQ (Storing)	Control	14.188	5.764	1.441	0.406	114.000	0.610	0.371
	Experimental	15.375	7.464	1.866	0.485			
WMQ (Attention)	Control	13.063	6.005	1.501	0.460	132.500	0.880	0.345
	Experimental	13.188	6.493	1.623	0.492			
WMQ (Executive)	Control	9.063	5.709	1.427	0.630	120.500	0.791	0.362
	Experimental	10.188	6.969	1.742	0.684			
WMQ (Full Scale)	Control	36.313	14.988	3.747	0.413	123.500	0.880	0.337
	Experimental	38.750	18.746	4.686	0.484			
WMI	Control	89.938	7.380	1.845	0.082	91.000	0.165	0.601
	Experimental	92.625	8.539	2.135	0.092			

Note: IADL-I = Instrumental activities of daily living—Independent; IADL-AR = Instrumental activities of daily living—Assistance Required; IADL-D = Instrumental activities of daily living—Dependent; WMQ = Working Memory Questionnaire; and WMI = Working Memory Index.

**Table 3:** Descriptive and comparative statistics post-test.

Variables	Group	Mean	SD	SE	C.V	*U*	S.S	BF_10_
IADL-I	Control	12.563	2.476	0.619	0.197	141.500	0.598	0.375
	Experimental	12.563	1.931	0.483	0.154			
IADL-AR	Control	0.900	1.197	0.379	1.330	24.500	0.088	0.992
	Experimental	1.889	1.453	0.484	0.769			
IADL-D	Control	1.750	2.315	0.818	1.323	19.000	0.939	0.483
	Experimental	1.200	1.095	0.490	0.913			
WMQ (Storing)	Control	12.438	6.470	1618	0.520	128.000	0.999	0.337
	Experimental	12.438	7.711	1928	0.620			
WMQ (Attention)	Control	9.250	6.191	1548	0.669	109.000	0.485	0.412
	Experimental	11.125	6.292	1573	0.566			
WMQ (Executive)	Control	8.125	5.353	1338	0.659	137.500	0.734	0.353
	Experimental	7.938	5.285	1321	0.666			
WMQ (Full Scale)	Control	29.813	15.510	3878	0.520	126.500	0.970	0.341
	Experimental	31.500	18.221	4555	0.578			
WMI	Control	93.688	7.040	1760	0.075	63.500	0.015	2.412
	Experimental	103.938	11.509	2877	0.111			

Note: IADL-I = Instrumental activities of daily living—Independent; IADL-AR = Instrumental activities of daily living—Assistance Required; IADL-D = Instrumental activities of daily living—Dependent; WMQ = Working Memory Questionnaire; and WMI = Working Memory Index.

**Table 4:** Comparative tests of related samples (pre-test–post-test) in the experimental group.

Variables	Mean	S.D	C.V.	W	z	*p*	BF_10_
IADL-I-PRE	9.688	3.516	0.363	9.500	−2.699	0.007	34.076
IADL-I-POS	12.563	1.931	0.154				
IADL-AR-PRE	3.000	3.651	1.217	17.000	−0.140	0.943	0.326
IADL-AR-POS	1.889	1.453	0.769				
IADL-D-PRE	1.313	1.537	1.171	6.000	1.604	0.174	1.439
IADL-D-POS	1.200	1.095	0.913				
WMQ (Storing) PRE	15.375	7.464	0.485	98.000	2.158	0.033	3.827
WMQ (Storing) POS	12.438	7.711	0.620				
WMQ (Attention) PRE	13.188	6.493	0.492	85.000	1.420	0.163	0.856
WMQ (Attention) POS	11.125	6.292	0.566				
WMQ (Executive) PRE	10.188	6.969	0.684	88.500	1.619	0.109	1.291
WMQ (Executive) POS	7.938	5.285	0.666				
WMQ (Full Scale) PRE	38.750	18.746	0.484	104.500	1.887	0.062	2.279
WMQ (Full Scale) POS	31.500	18.221	0.578				
WMI-PRE	92.625	8.539	0.092	3.000	−3.361	<0.001	180.797
WMI- POS	103.938	11.509	0.111				

Note. IADL-I = Instrumental activities of daily living—Independent; IADL-AR = Instrumental activities of daily living—Assistance Required; IADL-D = Instrumental activities of daily living—Dependent; WMQ = Working Memory Questionnaire; and WMI = Working Memory Index.

**Table 5:** Comparative tests of related samples (pre-test–post-test) in the active control group.

Variables	Mean	S.D	C.V.	W	z	*p*	BF_10_
IADL-I-PRE	10.188	3.619	0.355	0.000	−3.059	0.002	134.676
IADL-I-POS	12.563	2.476	0.197				
IADL-AR-PRE	2.688	2.938	1.093	21.000	2.201	0.035	6.687
IADL-AR-POS	0.900	1.197	1.330				
IADL-D-PRE	1.125	1.893	1.683	6.000	0.365	0.854	0.398
IADL-D-POS	1.750	2.315	1.323				
WMQ (Storing) PRE	14.188	5.764	0.406	78.000	1.601	0.116	1.147
WMQ (Storing) POS	12.438	6.470	0.520				
WMQ (Attention) PRE	13.063	6.005	0.460	111.500	2.925	0.004	77.418
WMQ (Attention) POS	9.250	6.191	0.669				
WMQ (Executive) PRE	9.063	5.709	0.630	88.000	1.034	0.312	0.383
WMQ (Executive) POS	8.125	5.353	0.659				
WMQ (Full Scale) PRE	36.313	14.988	0.413	102.000	2.385	0.018	3.357
WMQ (Full Scale) POS	29.813	15.510	0.520				
WMI-PRE	89.938	7.380	0.082	6.500	−2.550	0.012	21.669
WMI- POS	93.688	7.040	0.075				

Note. IADL-I = Instrumental activities of daily living—Independent; IADL-AR = Instrumental activities of daily living—Assistance Required; IADL-D = Instrumental activities of daily living—Dependent; WMQ = Working Memory Questionnaire; and WMI = Working Memory Index.

**Table 6:** Bayesian repeated-measures ANOVA for analyzing the effect of the depression variable.

Group	Likely Model	P(M|Data)	Time Effect (BF_Incl)	DYT Effect (BF_Incl)	Mean R^2^ (95% CI)
Experimental	Time	0.506	17.237 (Very Strong)	0.860 (Weak)	0.406 [0.197–0.578]
Control	Time + DYT	0.656	14.531 (Very Strong)	2.314 (Moderate)	0.580 [0.319–0.754]

Note: DYT = Depression Yesavage Test.

**Table 7:** Bayesian repeated-measures ANOVA for analyzing the effect of performance in MoCA Test.

Group	Likely Model	P(M|Data)	Time Effect (BF_Incl)	MOCA TOT Effect (BF_Incl)	Mean R^2^ (95% CI)
Experimental	Time + MOCA TOT	0.534	628.129 (Very strong)	1.149 (Anecdotical)	0.692 [0.518–0.812]
Control	Time + MOCA TOT	0.779	11.688 (Strong)	5.559 (Moderate to strong)	0.717 [0.433–0.857]

## Data Availability

The dataset used in this research is owned by Instituto Neurológico de Colombia (Colombia), and as such, its accessibility is subject to the institution’s policies and regulations. For this reason, the data is available upon request, as any data sharing must be approved by the university.

## References

[1] Feigin V.L., Brainin M., Norrving B., Martins S.O., Pandian J., Lindsay P., Grupper M.F., Rautalin I. (2025). World Stroke Organization: Global Stroke Fact Sheet 2025. Int. J. Stroke.

[2] Martin S.S., Aday A.W., Allen N.B., Almarzooq Z.I., Anderson C.A.M., Arora P., Avery C.L., Baker-Smith C.M., Bansal N., Beaton A.Z. (2025). 2025 Heart Disease and Stroke Statistics: A Report of US and Global Data From the American Heart Association. Circulation.

[3] El Husseini N., Katzan I.L., Rost N.S., Blake M.L., Byun E., Pendlebury S.T., Aparicio H.J., Marquine M.J., Gottesman R.F., Smith E.E. (2023). Cognitive Impairment After Ischemic and Hemorrhagic Stroke: A Scientific Statement From the American Heart Association/American Stroke Association. Stroke.

[4] Elendu C., Amaechi D.C., Elendu T.C., Ibhiedu J.O., Egbunu E.O., Ndam A.R., Ogala F.M., Ologunde T.M., Peterson J.C.M., Boluwatife A.I.M. (2023). Stroke and cognitive impairment: Understanding the connection and managing symptoms. Ann. Med. Surg..

[5] Blomgren C., Samuelsson H., Blomstrand C., Jern C., Jood K., Claesson L. (2019). Long-term performance of instrumental activities of daily living in young and middle-aged stroke survivors—Impact of cognitive dysfunction, emotional problems and fatigue. Abete P, editor. PLoS ONE.

[6] Stolwyk R.J., Mihaljcic T., Wong D.K., Chapman J.E., Rogers J.M. (2021). Poststroke Cognitive Impairment Negatively Impacts Activity and Participation Outcomes. Stroke.

[7] de Menezes K.K.P., Scianni A.A., Avelino P.R., Faria-Fortini I., Bastos V.S., de Morais Faria C.D.C. (2025). Contextual and clinical factors as explainers of stroke severity, residual motor impairments, and functional independence during hospitalization. J. Stroke Cerebrovasc. Dis..

[8] Heldner M.R., Chalfine C., Houot M., Umarova R.M., Rosner J., Lippert J., Gallucci L., Leger A., Baronnet F., Samson Y. (2022). Cognitive Status Predicts Return to Functional Independence After Minor Stroke: A Decision Tree Analysis. Front. Neurol..

[9] Mancuso M., Iosa M., Abbruzzese L., Matano A., Coccia M., Baudo S., Benedetti A., Gambarelli C., Spaccavento S., Ambiveri G. (2023). The impact of cognitive function deficits and their recovery on functional outcome in subjects affected by ischemic subacute stroke: Results from the Italian multicenter longitudinal study CogniReMo. Eur. J. Phys. Rehabil. Med..

[10] Lugtmeijer S., Lammers N.A., de Haan E.H.F., de Leeuw F.E., Kessels R.P.C. (2021). Post-Stroke Working Memory Dysfunction: A Meta-Analysis and Systematic Review. Neuropsychol. Rev..

[11] Irfani Fitri F., Fithrie A., Rambe A.S. (2020). Association between working memory impairment and activities of daily living in post-stroke patients. Med. Glas..

[12] Malouin F., Belleville S., Richards C.L., Desrosiers J., Doyon J. (2004). Working memory and mental practice outcomes after stroke. Arch. Phys. Med. Rehabil..

[13] Loetscher T., Potter K.J., Wong D., das Nair R. (2019). Cognitive rehabilitation for attention deficits following stroke. Cochrane Database Syst. Rev..

[14] Loetscher T., Lincoln N.B. (2013). Cognitive rehabilitation for attention deficits following stroke. Cochrane Database Syst. Rev..

[15] Westerberg H., Jacobaeus H., Hirvikoski T., Clevberger P., Östensson M.L., Bartfai A., Klingberg T. (2007). Computerized working memory training after stroke–A pilot study. Brain Inj..

[16] van de Ven R.M., Murre J.M.J., Veltman D.J., Schmand B.A. (2016). Computer-Based Cognitive Training for Executive Functions after Stroke: A Systematic Review. Front. Hum. Neurosci..

[17] Das Nair R., Cogger H., Worthington E., Lincoln N.B. (2016). Cognitive rehabilitation for memory deficits after stroke. Cochrane Database Syst. Rev..

[18] Kleim J.A., Jones T.A. (2008). Principles of Experience-Dependent Neural Plasticity: Implications for Rehabilitation After Brain Damage. J. Speech Lang. Hear. Res..

[19] Karbach J., Verhaeghen P. (2014). Making Working Memory Work: A Meta-Analysis of Executive-Control and Working Memory Training in Older Adults. Psychol. Sci..

[20] von Bastian C.C., Eschen A. (2016). Does working memory training have to be adaptive?. Psychol. Res..

[21] Cahn-Weiner D.A., Boyle P.A., Malloy P.F. (2002). Tests of Executive Function Predict Instrumental Activities of Daily Living in Community-Dwelling Older Individuals. Appl. Neuropsychol..

[22] Tucker A.M., Stern Y. (2011). Cognitive Reserve in Aging. Curr. Alzheimer Res..

[23] Simons D.J., Boot W.R., Charness N., Gathercole S.E., Chabris C.F., Hambrick D.Z., Stine-Morrow E.A.L. (2016). Do “Brain-Training” Programs Work?. Psychol. Sci. Public Interest.

[24] Lövdén M., Bäckman L., Lindenberger U., Schaefer S., Schmiedek F. (2010). A theoretical framework for the study of adult cognitive plasticity. Psychol. Bull..

[25] Robertson I.H., Murre J.M.J. (1999). Rehabilitation of brain damage: Brain plasticity and principles of guided recovery. Psychol. Bull..

[26] Lawton M.P., Brody E.M. (1969). Assessment of older people: Self-maintaining and instrumental activities of daily living. Gerontologist.

[27] Vallat-Azouvi C., Pradat-Diehl P., Azouvi P. (2012). The Working Memory Questionnaire: A scale to assess everyday life problems related to deficits of working memory in brain injured patients. Neuropsychol. Rehabil..

[28] Wechsler D. (2008). Wechsler Adult Intelligence Scale–Fourth Edition (WAIS–IV) Manual.

[29] Nasreddine Z.S., Phillips N.A., Bédirian V., Charbonneau S., Whitehead V., Collin I., Cummings J.L., Chertkow H. (2005). The Montreal Cognitive Assessment, MoCA: A brief screening tool for mild cognitive impairment. J. Am. Geriatr. Soc..

[30] Yesavage J.A., Brink T.L., Rose T.L., Lum O., Huang V., Adey M., Leirer V.O. (1982). Development and validation of a geriatric depression screening scale: A preliminary report. J. Psychiatr. Res..

[31] Faul F., Erdfelder E., Lang A.G., Buchner A. (2007). G*Power 3: A flexible statistical power analysis program for the social, behavioral, and biomedical sciences. Behav. Res. Methods.

[32] Jeffreys H. (1961). Theory of Probability.

[33] Maier M., Ballester B.R., Verschure P.F.M.J. (2019). Principles of neurorehabilitation after stroke based on motor learning and brain plasticity mechanisms. Front. Syst. Neurosci..

[34] Melby-Lervåg M., Redick T.S., Hulme C. (2016). Working memory training does not improve performance on measures of intelligence or other measures of “far transfer”: Evidence from a meta-analytic review. Perspect. Psychol. Sci..

[35] Mulhern M. (2023). Cognitive rehabilitation interventions for post-stroke populations. Delaware J. Public Health.

[36] Soni A.K., Kumar M., Kothari S. (2025). Efficacy of home-based computerized adaptive cognitive training in patients with post-stroke cognitive impairment: A randomized controlled trial. Sci. Rep..

